# Fluid dynamics of cerebrospinal fluid flow in perivascular spaces

**DOI:** 10.1098/rsif.2019.0572

**Published:** 2019-10-23

**Authors:** John H. Thomas

**Affiliations:** Department of Mechanical Engineering, University of Rochester, Rochester, NY 14627, USA

**Keywords:** cerebrospinal fluid, perivascular spaces, glymphatic system, fluid dynamics, diffusion, peristaltic pumping

## Abstract

The flow of cerebrospinal fluid along perivascular spaces (PVSs) is an important part of the brain’s system for delivering nutrients and eliminating metabolic waste products (such as amyloid-β); it also offers a pathway for the delivery of therapeutic drugs to the brain parenchyma. Recent experimental results have resolved several important questions about this flow, setting the stage for advances in our understanding of its fluid dynamics. This review summarizes the new experimental evidence and provides a critical evaluation of previous fluid-dynamic models of flows in PVSs. The review also discusses some basic fluid-dynamic concepts relevant to these flows, including the combined effects of diffusion and advection in clearing solutes from the brain.

## 1. Introduction

**Figure 1.**
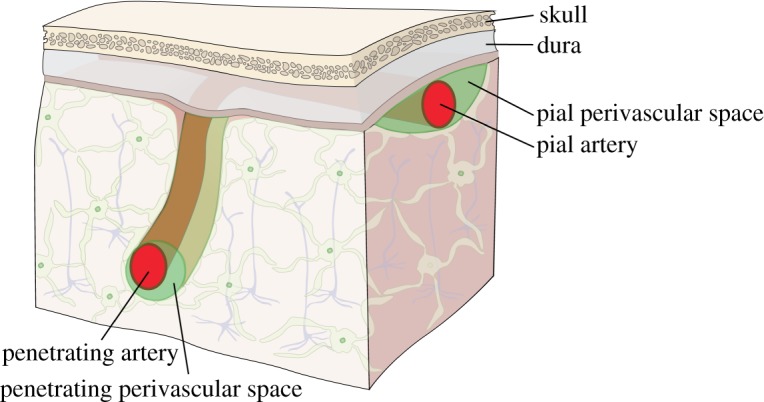
Schematic diagram of the PVSs around a pial artery and a penetrating artery in the brain. (Online version in colour.)

Perivascular spaces (PVSs) in the brain are channels that surround the blood vessels (e.g. arteries and veins) and are filled with cerebrospinal fluid (CSF). Figure 1 illustrates two such PVSs, those surrounding a pial artery and a penetrating artery, for which we have the best experimental data. It has long been thought that a flow of CSF in these PVSs plays an important role in the clearance of solutes from the brain [1–4]. In a seminal paper, Cserr *et al.* [5] showed that molecules of different molecular weight are cleared from the rodent brain at the same exponential decay rate, indicating bulk flow rather than diffusion as the dominant clearance mechanism. Subsequent experiments have shown that tracers injected into the subarachnoid space are transported preferentially through periarterial spaces at rates much faster than could be explained by diffusion alone [6–8]. Various mechanisms have been proposed as the driving force for this flow: an overall pressure gradient created by the production of CSF in the choroid plexuses; a pressure gradient driven by respiration [9]; or a peristaltic flow driven by arterial pulsations due to the heartbeat [6,10], a mechanism appropriately named *perivascular pumping* by Hadaczek *et al.* [11].

The clearance of metabolic proteins, such as amyloid-β, is an important process in the healthy brain: the failure of this process may be a cause of neurological disorders such as Alzheimer’s disease [12]. Many potentially effective drugs for the treatment of brain disorders do not readily cross the blood–brain barrier but can be delivered directly to the brain by injection under pressure through a small hole in the skull, a process known as convection-enhanced delivery (CED: see, for example, [13]). Following the injection, further spreading of the drug through the brain parenchyma is aided by the natural flow of CSF through PVSs and by diffusion. Understanding these transport processes is necessary for predicting the spatial distribution of infused drugs in the brain.

Recent experimental results, to be discussed below, have cleared up several important questions concerning the nature of flows of CSF in PVSs, thus providing a much firmer basis for future modelling of these flows. This is an opportune time to assess our experimental knowledge of these flows and to review and critique the theoretical work that has been done to understand and model these flows. That is the purpose of this review.

The flow of CSF in PVSs is an important part of the brain’s overall waste clearance system [14], sometimes known as the *glymphatic system* [15,16], which also includes the exchange of fluid between PVSs and the interstitial space in the brain through aquaporin-4 channels at the endfeet of astroglial cells or open spaces in the PVS wall. Here, I am using the term PVS to denote the space containing CSF surrounding any part of the vascular network, including arteries, arterioles, capillaries, venules and veins: these spaces include the Virchow–Robin spaces around arterioles and the basement membranes around capillaries. A challenging, long-term goal is to develop a comprehensive computational model of the fluid dynamics of this entire network. Ideally, this comprehensive model should have predictive capabilities, such that the effects of various pathologies or interventions (such as CED) can be predicted with some confidence. We are a long way from such a model, however: experiments have yet to reveal the details of flows in PVSs around penetrating arteries and in other regions in the parenchyma. In the near term, reasonable goals would be to develop a satisfactory computational model of the flow observed, in detail, in the PVSs around pial arteries in mice, and to develop a relatively simple hydraulic network model (or lumped-parameter model) of flows and diffusion in the entire glymphatic system. Progress towards these goals is reviewed here.

This review focuses rather narrowly on the fluid dynamics of the flow of CSF in PVSs and on the relative roles of advection and diffusion in these flows, with an emphasis on the implications of recent experimental results. For a broader view of the fluid dynamics of CSF and related clinical issues, see the reviews by Kurtcuoglu [17], Brinker *et al.* [14] and Linninger *et al.* [18]; for a more general discussion of the role of diffusion, see the review by Nicholson & Hrabětová [19].

## 2. Recent experimental results

### 2.1. Perivascular pumping

Recent *in vivo* experiments with mice show unequivocally that there is pulsatile flow in the PVSs around arteries in the brain [20,21], with net (bulk) flow in the same direction as the blood flow [21], and that this flow is largely driven by the cardiac cycle [21]. From the continuity equation (expressing conservation of mass), we know that this net flow must continue in some form through other parts of the system (e.g. PVSs around arterioles, capillaries, venules). The *in vivo* experimental methods of Mestre *et al.* [21] now enable detailed measurements of the actual size and shape of the PVSs, the motions of the arterial wall and the velocity field of the flow of CSF.

These recent results have cleared up a number of questions about flows in PVSs and provide a firm basis for studying the fluid dynamics of perivascular pumping through computational models. We now have good estimates of the values of several input parameters and measured flow velocities to compare with the outputs of models.

**Figure 2.**
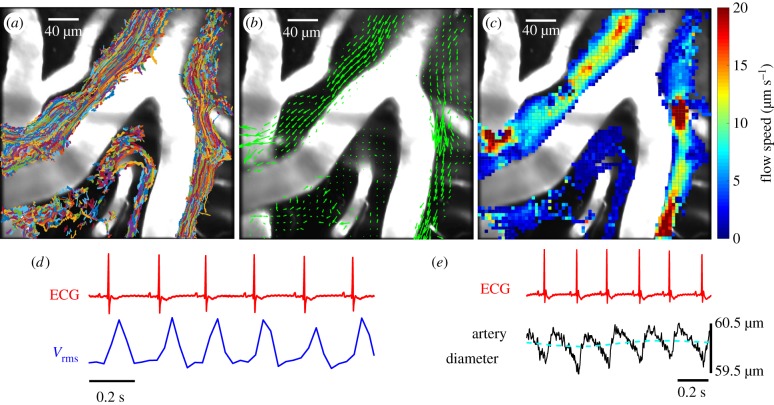
Periarterial flow in mouse pial arteries, as revealed by particle tracking (from Mestre *et al.* [21]). (*a*) Superimposed trajectories of tracked microspheres. (*b*) Time-averaged velocity field (green arrows), showing net flow in the direction of the blood flow. (*c*) Time-averaged flow speed. (*d*) Representative traces of the ECG and the root-mean-square flow velocity *V*_rms_, showing synchrony with the heartbeat. (*e*) Representative traces of the ECG and the artery diameter, showing the shape of the wall wave driving the perivascular pumping. (Online version in colour.)

The following new experimental results of Mestre *et al.* [21], based on particle-tracking velocimetry (figure 2), have particular implications for the fluid dynamics of PVSs.

1. The flow of CSF in the PVS around near-surface pial arteries is pulsatile, driven primarily by oscillations of the artery wall due to the heartbeat, with net flow in the direction of the blood flow. These results contradict claims that arterial pulsations are too weak to drive a net flow [22,23] or that the net flow is in the direction opposite to that of the blood flow [24]. The results also show that respiration is at most a modulating factor, not the primary driver of the flow.
2. The shape of the arterial wall wave driving the flow is not sinusoidal, as assumed in many models of perivascular pumping. The non-sinusoidal shape seems to be essential. In normal mice, each cardiac cycle involves a fast expansion of the artery wall, followed by a slow contraction. The fluid speed matches: fast flow, then slower. In mice with acute hypertension, each cardiac cycle involves a fast expansion, a fast contraction and then slow contraction, and there is significant backflow and reduced net flow, probably due to the fast contraction.
3. The PVSs around pial arteries, as measured *in vivo*, are much larger than previous estimates based on electron microscope images of fixed tissue. As such, the PVS offers much less viscous resistance to flow of CSF than previously thought.
4. The cross-section of the PVS around a pial or penetrating artery is generally quite different from the circular annulus usually assumed in models: it is flattened, often two-lobed and sometimes eccentric. As such, it offers less viscous resistance to the flow, as demonstrated in a recent paper [25] (to be discussed below).
5. The PVS around an artery is not a porous medium: the parabolic-like velocity profiles, the smooth particle tracks and the collapse during fixation indicate that the PVS is mostly an unobstructed, fluid-filled space. Hence, the flow of CSF in these spaces is best described by the Navier–Stokes equation rather than the Darcy–Brinkman equation.

In this review, I discuss basic fluid-dynamic theory as it applies to perivascular flows, some published models of perivascular pumping and hydraulic network models of the entire glymphatic system and the role of diffusion in combination with the flow of CSF.

### 2.2. The size and shape of the periarterial space

In order to model perivascular pumping in the PVS around an artery, we need to know the size and shape of the PVS itself. The recent *in vivo* measurements of periarterial flows in mouse brain by Mestre *et al.* [21] reveal the true size and shape of PVSs around pial arteries. Figure 2*a* shows a superposition of all the particle tracks in the PVS around a pial artery from a single experiment: these tracks effectively trace out the full extent of the PVS and show that the width of the PVS is comparable to the width of the artery itself. Other *in vivo* experiments with injected dye show similar results [20,25,26]. The size of the PVS is substantially larger than that shown in previous electron microscope measurements of fixed tissue. Mestre *et al.* show in detail (including a movie) how the PVS collapses to about a tenth of its cross-sectional area during fixation. These results show that PVSs are much larger in the live brain and point out the importance of studying their configuration *in vivo*.

The discovery of the large size of the PVS helps to clear up one problem. Some hydraulic network models of the flow of fluid in the brain have concluded that any significant flow is unlikely because of the high hydraulic resistance of the system. However, these studies, which have generally taken the cross-section of the PVS to be in the form of a concentric circular annulus, have assumed much smaller widths of the annulus than observed *in vivo*. For a circular annulus with inner and outer radii *r*_1_ and *r*_2_, respectively, for fixed *r*_1_ the hydraulic resistance scales roughly as (*r*_2_/*r*_1_)^−4^, and hence is greatly reduced in a wider annulus. Based on the sizes of arterial PVSs measured by Mestre *et al.*, the actual hydraulic resistance is significantly lower than values assumed in some published models. Moreover, *in vivo* observations of the cross-sections of PVSs around pial and penetrating arteries reveal that they are far from being concentric circular annuli: they are usually more flattened, or elliptical, or not concentric with the artery they surround. This result has significant implications, which I will discuss below.

## 3. Some basic fluid dynamics

Here I review, in general terms, some elementary fluid dynamics, as applied to flows in PVSs. Normal CSF is mostly water and behaves as a Newtonian viscous fluid [27], meaning that in a shearing flow the shear stress is proportional to the strain rate. In this sense, flows in the PVS are simpler to analyse than the flow of blood, which is non-Newtonian because its resistance to shearing is reduced at higher strain rates, as the suspended blood cells and proteins become elongated and aligned with the flow. The viscosity of normal CSF, which contains only a small amount of solutes or suspended particles, differs little from that of pure water.

In the study of perivascular flows, we are dealing with the flow of an incompressible, Newtonian viscous fluid in an essentially open (non-porous) space. The equation of motion is the Navier–Stokes equation

3.1 $$\rho \left(\frac{\partial u}{\partial t}+u\cdot \nabla u\right)=-\nabla p+\mu \nabla^{2}u,$$

where **u** is the Eulerian velocity field (referred to a fixed frame of reference), *ρ* is the fluid density, *p* is the fluid pressure, *μ* is the dynamic viscosity, *t* is the time, ∇ is the spatial gradient operator and $\nabla^{2}$ is the Laplacian operator: in Cartesian coordinates, ∇=(∂/∂x,∂/∂y,∂/∂z) and $\nabla^{2}=\nabla \cdot \nabla =\partial^{2}/\partial x^{2}+\partial^{2}/\partial y^{2}+\partial^{2}/\partial z^{2}$. In addition, we have the continuity equation expressing conservation of mass, which for an incompressible fluid is

3.2 ∇⋅u=0.

We can assume that the density *ρ* and viscosity *μ* are constant and uniform. Then the vector equation (3.1) and the scalar equation (3.2) form a closed set of four scalar equations for the four scalar unknowns: the pressure *p* and the three scalar components of the velocity field **u** = (*u*, *v*, *w*). These equations must be supplemented by the appropriate boundary conditions. For example, at a solid, impenetrable wall, there is no flow across the wall, and no flow along the wall (the no-slip condition for a viscous fluid), so the velocity of the fluid must match the velocity of the wall at that boundary. For a wall at rest, we must have **u** = 0 at the wall.

Dimensional analysis plays a central role in fluid dynamics. There are numerous cases where it is helpful to compare the magnitude of two different terms in an equation by forming their dimensionless ratio. The most important example is the *Reynolds number*, which estimates the ratio of the nonlinear inertial term ρu⋅∇u and the viscous term $\mu \nabla^{2}u$ in the Navier–Stokes equation. Let *U* be a scale for the magnitude of the velocity, |**u**|, and let *L* be a length scale for variations in the velocity. The Reynolds number is then defined as

3.3 $$\frac{\left|\rho u\cdot \nabla u\right|}{\left|\mu \nabla^{2}u\right|}∼\frac{\rho U(U/L)}{\mu (U/L^{2})}∼\frac{\rho UL}{\mu }=\frac{UL}{\nu }\equiv Re,$$

where *ν* = *μ*/*ρ* is the kinematic viscosity. For perivascular flows, the Reynolds number is very small, *Re* ≪ 1, so the nonlinear term u⋅∇u in the Navier–Stokes equation can be safely neglected. (For example, for the flows we observed in the mouse periarterial space [21], *Re* ∼ 10^−3^, and it is likely that *Re* ≪ 1 in human PVSs as well.) Thus, perivascular flows are part of a large class of viscous-dominated flows in which inertial effects are negligible: this class includes flows associated with lubricating oil in wheel bearings and the swimming of micro-organisms.^1^ We can neglect the nonlinear term in the Navier–Stokes equation, and this simplifies the analysis considerably. However, there are still several other complicating factors in perivascular flows. The flow is time dependent, and the shape of the flow channel is irregular. The boundary conditions are tricky: the walls are compliant, and perhaps porous, and are moving.

## 4. Models of perivascular pumping driven by arterial pulsations

There have been a few studies involving theoretical models of flows in the PVS driven by arterial pulsations, which have reached conflicting conclusions. I review these studies here, and, in a couple of cases, point out serious difficulties with the models.

The computational study by Bilston *et al.* [28] investigates CSF flow, driven by arterial pulsations, along PVSs within the spinal cord. They model the cross-section of the PVS as a circular annulus surrounding an artery: this space is non-porous and filled with a Newtonian fluid. Systolic pulsations are modelled as waves (with a Gaussian shape) travelling along the arterial wall. They find that, in the absence of an overall imposed pressure gradient, these pulsations drive flow in both directions, but with net (time-averaged) flow in the direction of the moving wave, i.e. in the direction of the blood flow. Unfortunately, the results they present are not relevant to the problem at hand because they used impossibly small wavelengths, of the order of 200 μm, in their calculations, corresponding to pulse rates of the order of 25 000 Hz for their assumed propagation speed of 5 m s^−1^. A wave speed of the order of 1 m s^−1^ is often assumed in models, but this quantity does not seem to have been actually measured. (For a discussion of arterial pulse speed measurements and models, see Reymond *et al.* [29].) For a wave speed of 1 m s^−1^ and a pulse rate of 300 beats per minute (for mice), the wavelength of the arterial wall wave is 20 cm. This wavelength is much longer than the width of the perivascular flow channel, which leads to an important simplification of the governing flow equations [30]. (This wavelength is also much longer than the length of the perivascular channels, but approximating the wave as a standing wave, as is sometimes done, loses essential physics. This point will come up again below.)

Schley *et al.* [24] present a two-dimensional Cartesian model of the PVS as a thin layer (100–150 nm thick) between the artery wall and the brain. Both boundaries are allowed to be permeable. The viscous-dominated flow is driven by pulse-wave deformations of the arterial boundary. They find analytical solutions for the instantaneous volume flux rate *q* and the time-averaged flux rate over a pulse period. For impermeable boundaries, they find that there are instantaneous flows in both the positive and negative axial directions, but the time-averaged flow is always in the direction of propagation of the pulse wave. However, based on their own tracer studies, Schley *et al.* seek to explain reverse transport, in the opposite direction of the blood flow. Because there is some instantaneous flux in the negative *x*-direction, they suggest that an ‘attachment mechanism’, whereby a solute attaches and then releases from a boundary wall, might produce a valve-like effect, leading to reverse transport. As an alternative, they suggest a mechanism in which the channel becomes very narrow during the time when the pressure gradient is negative, slowing the forward flow. In a related paper [31], yet another mechanism for reverse flow is postulated: flexible structures within the basement membrane that orient themselves to present greater hydraulic resistance to forward flow than to reverse flow. None of these mechanisms has any experimental support; in view of the recent experimental confirmation of forward net flow, no such mechanism is necessary. Coloma *et al.* [32] also tried to demonstrate reverse transport in peristaltic flow. They suggest that a reverse transport in the blood vessel wall (the arterial basement membrane) might be driven by arterial pulsations provided that there are strong reflections of the pulse waves at branch points in the arteries. However, since both Schley *et al.* and Coloma *et al.* find that arterial pulsations drive a net flow in the same direction as the blood flow in normal circumstances, their analyses actually agree with the recent finding of forward transport if one omits the hypothetical mechanisms for reverse transport that they propose.

The most relevant theoretical model of perivascular pumping, in light of recent experimental findings, is that of Wang & Olbricht [33]. They present a circular annulus model in which the PVS is taken to be porous, and hence they adopt the Darcy term rather than the usual viscous term in the equation of motion. They find an analytical solution based on the low Reynolds number, long wavelength approximation [30,34], which is well suited to PVS flows. They find that the time-averaged volume flow is always in the direction of propagation of the arterial pulse wave, i.e. in the direction of the blood flow. Their solution for the time-averaged volume flow rate *Q* is the following:

4.1 $$\begin{array}{rl} Q & =\pi r_{2}^{2}\epsilon c\left(\frac{2\alpha^{2}}{1-\alpha^{2}}\right){\left(\frac{b}{r_{2}}\right)}^{2}+\pi r_{2}^{2}(1-\alpha^{2})\left(-\frac{\kappa \Delta p_{\lambda }}{\lambda \mu }\right)\\ & \;-\pi r_{2}^{2}\left(\frac{1+3\alpha^{2}}{2(1-\alpha^{2})}\right)\left(-\frac{\kappa \Delta p_{\lambda }}{\lambda \mu }\right){\left(\frac{b}{r_{2}}\right)}^{2}, \end{array}$$

where *α* = *r*_1_/*r*_2_ is the ratio of the inner and outer radii of the circular annulus, *μ* is the dynamic viscosity, *ε* is the porosity of the PVS, *κ* is the Darcy permeability, $\Delta p_{\lambda }$ is the pressure drop over one wavelength *λ*, *b* is the amplitude of the wall wave and *c* is the propagation speed of the wall wave. The net volume flow rate is thus the sum of the three terms on the right-hand side of this equation. The first term represents the net flow due to peristaltic pumping by a small-amplitude wall wave in the absence of an overall pressure gradient ($\Delta p_{\lambda }=0$). The second term represents the net flow due to an overall pressure gradient. The third term represents a coupling between the effects of the wall wave and the overall pressure gradient, accounting for the fact that the pressure-driven flow moves through the annulus whose shape is distorted by the wall wave: for small wave amplitudes (*b*/*r*_2_ ≪ 1) this term is negligibly small. Note that the first term on the right, representing peristaltic pumping, does not involve the viscosity: it is a purely geometric term representing the squeezing effect of the wall wave on the incompressible fluid. This term, with *ε* = 1, is the same as one would obtain for flow in an open (non-porous) space. It is perhaps curious that this term does not involve the viscosity, but of course the viscosity does come into play in determining (based on the second term) what pressure gradient would be necessary to cancel the net peristaltic flow, and the viscosity also affects the amplitude and propagation speed of the arterial wall wave driven by the heartbeat. Using typical parameter values from our mouse experiments [21] (*r*_1_ = 20 μm, *r*_2_ = 40 μm, *b* = 0.2 μm, $c=1\;\text{m}\;{\text{s}}^{-1}$) in the analytical expression (4.1) for the mean flow rate, for the case of a free space (*ε* = 1) and no overall pressure gradient ($\Delta p_{\lambda }=0$), gives average flow speeds of about 20 μm s^−1^, in good agreement with the measured average flow speeds and also comparable to the measured maximum lateral wall speed (about 21 μm s^−1^), indicating that peristaltic pumping is indeed the main driving force for the flow.

If we discount the attempts to include additional effects to explain a reverse net flow of CSF, all of the models of perivascular pumping mentioned above predict oscillating flows in the PVSs with net flow in the same direction as the blood flow, consistent with the measurements of Mestre *et al.* [21].

In 2018, Rey & Sarntinoranont [35] presented a model of an annular PVS with a surrounding porous region modelling the parenchyma and flow driven by pulsations of the arterial wall (inner wall of the annulus). They found that there is no net flow in either direction in their model. However, this result is an inevitable consequence of the fact that they take the wall motions to be in the form of a standing wave: as such, there is no preferred direction and consequently no net flow. Any net peristaltic flow in their model would require a travelling wall wave, as is indeed the case for arterial pulsations. As they correctly point out, because of the long wavelength of the wall wave there is only a small phase difference over the short length of the channel in their model; nevertheless, the propagation of the wave cannot be ignored. Note that the peristaltic pumping term (the first term on the right-hand side) in equation (4.1) is proportional to the high propagation speed *c* of the wall wave, multiplied by the very small amplitude factor (*b*/*r*_2_)^2^. In a recent paper, Coloma *et al.* [36] report on experiments with a microfluidic device that mimics perivascular pumping. They find a net flow in the same direction as that of the wall wave, even though the wavelength of the wall wave (about 1 m) is very much greater than the length of the flow channel (2 cm). (They also show that reflections of the wall wave can produce a reverse flow under special circumstances.)

## 5. Hydraulic network models and hydraulic resistance

A *hydraulic network model* of the flow through the system of PVSs is basically a branching network of uniform, one-dimensional ducts carrying the flow of CSF. Such an approach is supported by recent tracer studies that reveal that the connections between PVSs continue throughout the entire vascular system, from arteries to arterioles to capillaries to venules to veins, providing a route for CSF flow [37]. A hydraulic network model can incorporate additional efflux routes along nerve cells and into cerebral lymphatic vessels, for which there is also experimental evidence. The model can also include a flow of water across porous duct walls into a space corresponding to the parenchyma, representing the exchange of water (but not all solvents) through aquaporin-4 channels between CSF in a PVS and interstitial fluid (ISF) in the parenchyma, and a flow of ISF through the parenchyma, in accord with the glymphatic system [8].

A steady, laminar (Poiseuille) flow of fluid along a uniform duct is characterized by a volume flow rate *Q* that is proportional to the pressure drop Δ*p* along the channel and inversely proportional to a *hydraulic resistance* *R*, which can be calculated from the viscosity of the fluid, the shape and area of the cross-section of the duct and the length of the duct. Higher hydraulic resistance impedes flow, such that a smaller volume of CSF is pumped per second by a given pressure drop Δ*p*; lower hydraulic resistance promotes flow. Hydraulic resistance is analogous to electrical resistance, which impedes the electrical current (analogous to *Q*) driven by a given voltage drop (analogous to Δ*p*).

Hydraulic network models published in 2018 [35,38] assume the PVSs to have cross-sections in the form of a concentric circular annulus. These papers argue against any significant bulk flow of CSF because of high hydraulic resistance of the network. There are two significant problems with these models; however, they underestimate the size of the PVSs (thus overestimating the hydraulic resistance), and their assumed concentric circular annulus cross-sections offer the greatest hydraulic resistance for a given PVS cross-sectional area [25]. Here, I discuss these points further.

### 5.1. Hydraulic resistance for steady Poiseuille flow in a uniform annular duct

The hydraulic resistance of a particular model of a PVS cross-section can be determined by solving the equation for steady Poiseuille flow in that configuration. For this flow, we are concerned with the velocity distribution for steady, fully developed, laminar viscous flow in a uniform annular tube, driven by a uniform pressure gradient in the axial (*z*) direction. The velocity **u** = [0, 0, *u*(*x*, *y*)] is purely in the *z*-direction and the nonlinear term in the Navier–Stokes equation is identically zero. The basic partial differential equation to be solved is the *z*-component of the Navier–Stokes equation, which reduces to

5.1 $$\frac{\partial^{2}u}{\partial x^{2}}+\frac{\partial^{2}u}{\partial y^{2}}=\frac{1}{\mu }\frac{\text{d}p}{\text{d}z}=\text{constant},$$

where *μ* is the dynamic viscosity of the CSF and the pressure gradient d*p*/d*z* is constant (and negative for flow in the positive *z*-direction). This is Poisson’s equation with a constant inhomogeneous term on the right-hand side. We need to solve this equation subject to the Dirichlet (no-slip) condition *u* = 0 on the inner and outer boundaries of the annulus. Analytic solutions are known for simple geometries [39] (including a concentric circular annulus), and it is straightforward to calculate numerical solutions for more complicated geometries.

### 5.2. Hydraulic resistance for an improved model of PVSs

As mentioned, theoretical models of flow in PVSs have generally assumed that the PVS cross-section is in the form of a concentric circular annulus. In a recent paper, Tithof *et al.* [25] show that the actual observed shapes of PVSs have lower hydraulic resistance than concentric circular annuli of the same size, and therefore allow faster flow of CSF. Indeed, the shape of actual PVSs is very nearly optimal, in the sense of giving the least hydraulic resistance. The authors suggest that this is the result of evolutionary adaptation [25].

**Figure 3.**
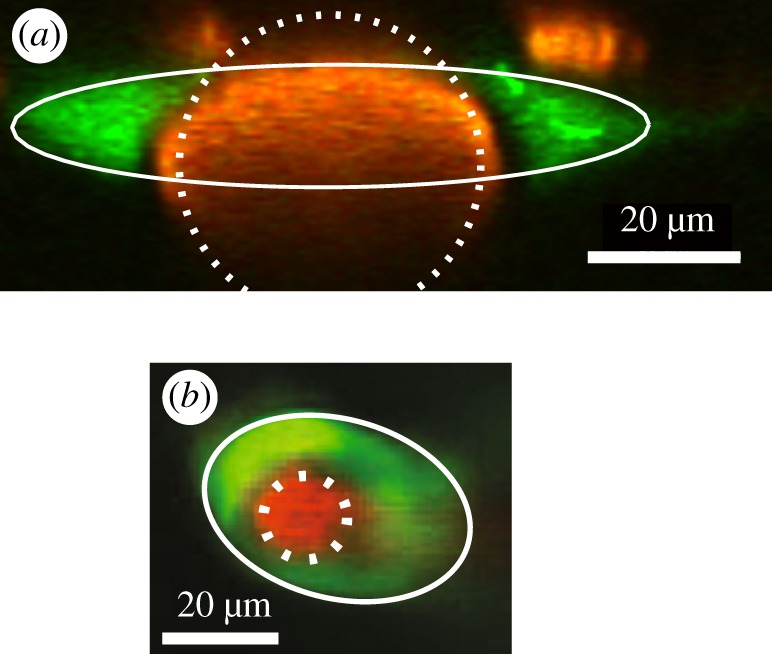
Examples of the cross-sections of PVSs (green) around arteries (red), observed *in vivo*. (*a*) Pial artery (from [21]). (*b*) Penetrating artery (adapted from [40]). The white lines show fits to a simple, adjustable geometric model of the cross-section of the PVS, consisting of an elliptical outer wall and a circular artery (from Tithof *et al.* [25]). (Online version in colour.)

Tithof *et al.* use an adjustable geometric model of the PVS cross-section that can be fitted to the various shapes actually observed. The cross-section is bounded by an inner circle, representing the outer wall of the artery, and an outer ellipse, representing the outer wall of the PVS. The radius of the circular artery, the semi-major and semi-minor axes of the ellipse, and the eccentricity of the ellipse relative to the circle can be varied to closely match different observed cross-sections of the PVSs. Figure 3 shows two examples (from [25]) of the fit of the model to the actual configuration of a PVS observed *in vivo* in a dye experiment. The hydraulic resistance of each configuration of the model cross-section is computed numerically by solving the Poisson equation (5.1) for that boundary. The minimum hydraulic resistance (and therefore maximum flow rate) for a given PVS cross-sectional area occurs for cases where the ellipse is flattened and intersects the circle and hence the PVS is divided into two disconnected lobes: this is a common configuration around pial arteries. The optimal shape reduces hydraulic resistance by placing more of the fluid away from the bounding walls, thus reducing the viscous shear stress in the flow. Further flattening of the ellipse narrows the channels and resistance begins to increase. When the inner and outer boundaries of the PVS are nearly circular, as is common around penetrating arteries, the minimum hydraulic resistance occurs when the eccentricity is large.

### 5.3. Hydraulic resistance and perivascular pumping

What is the relevance of the hydraulic resistance calculated for steady Poiseuille flow to the pulsatile flow in a PVS, driven by peristaltic pumping due to arterial wall motions? The perivascular pumping occurs mainly in the periarterial spaces around the proximal sections of the main cerebral arteries: at more distal locations the wall motions become smaller and the flow is driven mainly by the oscillating pressure gradient generated by the perivascular pumping upstream (as described by the second term on the right of equation (4.1) with an oscillating $\Delta p_{\lambda }$). Viscous, incompressible duct flows due to oscillating pressure gradients are well understood: the problem posed is linear, and analytical solutions are known for some simple duct cross-sections. The nature of the solution depends on the *dynamic Reynolds number* *R*_d_ = *ω*ℓ^2^/*ν*, where *ω* is the angular frequency of the oscillating pressure gradient, *ν* is the kinematic viscosity and ℓ is the length scale of the cross-section, e.g. the gap width for a circular annulus. (Alternatively, the *Womersley number* $\alpha =\sqrt{R_{d}}$ is often used in biofluid mechanics.) When *R*_d_ ≪ 1, as is in the case of flows in PVSs, the velocity profile is very nearly that corresponding to quasi-static Poiseuille flow in phase with the oscillating pressure gradient (see [39], §§3–4.2). In this case, the average volume flow rate is inversely proportional to exactly the same hydraulic resistance that applies to steady Poiseuille flow, and these hydraulic resistances will apply to PVSs throughout the network model, except for proximal sections of main arteries where the perivascular pumping is actually taking place.

In proximal periarterial spaces, where the perivascular pumping operates, the flow is actively driven by travelling wave motions of the arterial wall. With an elliptical outer boundary, we can expect the flow to be three-dimensional, with secondary motions in the azimuthal direction, even when the wall wave is axisymmetric. We can construct the following qualitative description of the flow based on what is known for the circular annulus. (This qualitative description is borne out by detailed calculations in a forthcoming paper [41].) According to equation (4.1), the effectiveness of the pumping scales as (*b*/ℓ)^2^, where *b* is the amplitude of the wall wave and ℓ is the width of the gap between the inner and outer boundaries. For a concentric circular annulus, the gap width ℓ and hence the pumping effectiveness are axisymmetric, so the resulting flow is also axisymmetric and the streamlines lie in planes through the central axis and wiggle in the radial direction. For an elliptical outer boundary, however, the gap width ℓ and, hence, the pumping effectiveness vary in the azimuthal direction, so there will be pressure variations in the azimuthal direction that drive a secondary, oscillatory flow in the azimuthal direction, and the streamlines will also wiggle in the azimuthal direction. Flattening the ellipse for a fixed area ratio will decrease the flow resistance but will also decrease the pumping efficiency because (i) more fluid is placed further from the artery wall and (ii) in cases where the PVS is split into two lobes only part of the artery wall is doing the pumping. Therefore, we might expect that there will be an optimal aspect ratio of the ellipse that will produce the maximum mean flow rate, and that this optimal ratio will be different from that which just produces the lowest hydraulic resistance. I have suggested that evolutionary adaptation has produced shapes of actual periarterial spaces around proximal sections of the main arteries that are nearly optimal in this sense [25].

## 6. Combined advection and diffusion

Diffusion is an important process in transporting metabolites and other solutes in the brain [19,42], and some have argued that it is the dominant process. But now that we have direct evidence of bulk CSF flow in periarterial spaces, in which advection dominates diffusion, it is appropriate to consider more generally the relative importance of advection and diffusion in transporting solutes. (Note that I am using the term advection, rather than convection, for the bulk transport of a solute by a flow.) I will also discuss how advection and diffusion act together to more effectively disperse a solute. Fluid dynamicists generally refer to the combined effects of advection and diffusion as *dispersion* (not convection, as sometimes used in the neuroscience literature).

A misconception about diffusion appears here and there in the literature, exemplified by the statement: ‘Diffusion is always occurring, whereas bulk flow requires a driving force, such as a pressure gradient’. Yes, net transport by advection requires a force to drive the flow, but net transport by diffusion requires a gradient in the concentration of the diffusing solute. If the concentration of the solute is spatially uniform, the solute molecules are, of course, undergoing random motions, but there is no net diffusive transport: over a fixed time interval, there are equal numbers of solute molecules crossing in either direction across any imaginary surface within the medium. In a closed system, with no creation of new solute, diffusion will eventually produce a uniform concentration of solute and no further net transport will occur: if there is creation of the solute, distributed uniformly, the concentration will remain uniform but increase with time. Some mechanism in the system is needed to maintain concentration gradients in order for net diffusive transport to continue. This mechanism may well involve advection by a bulk flow through parts of the system.

### 6.1. The advection–diffusion equation and the Péclet number

The concentration *C* of a conserved solute (with no sources or sinks) in a flowing fluid obeys the advection–diffusion equation

6.1 $$\frac{\partial C}{\partial t}+u\cdot \nabla C=D\nabla^{2}C,$$

where **u** is the Eulerian velocity and *D* is the diffusion coefficient for the solute. As a first approximation, we can consider a solute in normal CSF to be passive, in the sense that variations in its concentration have no effect on the flow. For a passive solute, the advection–diffusion equation is decoupled from the fluid-dynamic equations: one can compute a certain flow field independently, and then solve the advection–diffusion equation using the computed flow field. Thus, we have the possibility of studying advection–diffusion using results of computational flow models. At higher concentrations, a solute may not be passive: for example, it may alter the viscosity of the fluid and hence alter the flow field.

The relative importance of advection and diffusion is measured by a dimensionless number, the *Péclet number* *Pe*, which estimates the ratio of the advection and diffusion terms in the equation above:

6.2 $$\frac{\left|u\cdot \nabla C\right|}{\left|D\nabla^{2}C\right|}∼\frac{U(C/L)}{D(C/L^{2})}∼\frac{UL}{D}\equiv Pe,$$

where *U* is a velocity scale, *C* is a typical magnitude of the concentration and *L* is a length scale for variations in the concentration. Note the analogy between the Péclet number *Pe* = *UL*/*D* and the Reynolds number *Re* = *UL*/*ν*. Each is the product of a velocity scale and a length scale, divided by a diffusivity: for the Péclet number, the diffusivity is *D*, the diffusivity of the solvent; for the Reynolds number, the diffusivity is the kinematic viscosity *ν*, which can be considered as the diffusivity of momentum.

For the periarterial flows observed by Mestre *et al.* [21] in the mouse periarterial space, the Péclet number is large: *Pe* ∼ 1000 for the microspheres used in the experiments and *Pe* ∼ 10 − 100 for other solutes. Hence, in these perivascular flows advection dominates diffusion. We do not expect this to be the case throughout the entire glymphatic system, however, because of the branching of the flow into many channels.

Frequently in the literature we find statements such as the following: ‘diffusion is the dominant process at small scales (or, over short distances), whereas advection is the dominant process at large scales (or, over long distances)’. But this is not always true, as shown by the following example. Suppose we have laminar flow in a single pipe of inner diameter *d*. The Reynolds number is *Re* = *Vd*/*ν* and the Péclet number is *Pe* = *Vd*/*D*, where *V* is the average velocity over the cross-section of the pipe. Now suppose the pipe narrows (i.e. *d* decreases) downstream. The volume flux rate *Q* = *VA*, where *A* = *πd*^2^/4 is the cross-sectional area of the pipe, is constant along the pipe (conservation of mass), so *V* = *Q*/*A* ∼ *Q*/*d*^2^, and hence the flow speeds up as the pipe narrows and

$$Re=\frac{Vd}{\nu }∼\frac{Q}{d^{2}}\frac{d}{\nu }∼\frac{Q}{d\nu }\;\text{and}\;Pe=\frac{Vd}{D}∼\frac{Q}{d^{2}}\frac{d}{D}∼\frac{Q}{dD}$$

and we see that both *Re* and *Pe* increase as the pipe narrows. Hence, advection actually becomes more dominant over diffusion as the pipe narrows and the flow speeds up.

But, of course, the network of PVSs is far from being a single pipe! It may be thought of in simple terms as a complicated network of branching pipes. In a system of pipes branching from a single pipe, the entering volume flow rate *Q* = *VA* splits into *n* different pipes of volume flow rate *Q*_*i*_ and cross-sectional area *A*_*i*_, such that $Q=\sum_{i=1}^{n}Q_{i}=\sum_{i=1}^{n}V_{i}A_{i}=V_{\mathrm{av}}\sum_{i=1}^{n}A_{i}=V_{\mathrm{av}}A_{\mathrm{total}}$, where *V*_av_ is the average velocity for all *n* of the downstream pipes and *A*_total_ is the total cross-sectional area of all the downstream pipes. For *A*_total_ ≫ *A*, we have *V*_av_ ≪ *V*, that is, the flow speeds in the downstream pipes are greatly reduced compared with the beginning pipe, and diffusion becomes more important in the downstream pipes. We expect something like this to be the case in the glymphatic system as the flow in the PVS around an artery branches into numerous spaces around arterioles and then capillaries, and exchanges with ISF through aquaporin-4 channels [43].

### 6.2. Taylor dispersion

**Figure 4.**
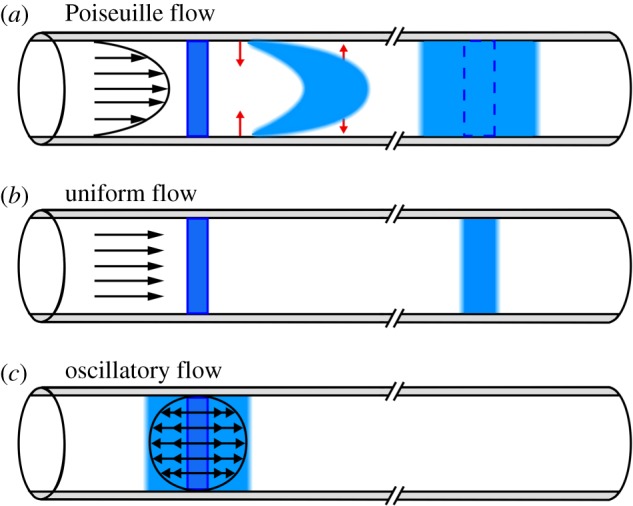
(*a*) Taylor dispersion of a solute (blue) in steady, laminar pipe flow (Poiseuille flow, with velocity profile indicated by the arrows). The solute is spread by the velocity shear, while diffusion in the radial direction (red arrows) establishes a uniform radial distribution. The velocity shear continues to spread out the solute further downstream. (*b*) For comparison, the effect of diffusion alone in a (non-physical) uniform flow (without shear). (*c*) Taylor dispersion in a purely oscillatory flow: the velocity shear spreads out the solute, but only to an extent limited by the fluid particle paths, and there is no downstream transport. (Online version in colour.)

The processes of advection and diffusion can combine to produce greatly enhanced dispersion of a solute in a shearing flow, as demonstrated every time you stir your coffee after adding some cream. In a viscous shearing flow along a narrow channel, the combination of advection and diffusion leads to an enhanced dispersion of solute in the axial direction, a mechanism known as Taylor dispersion (after Taylor [44]). To illustrate this mechanism, let us consider Taylor dispersion in steady, laminar (Poiseuille) flow in a narrow pipe, driven by an axial pressure gradient. This flow has a parabolic velocity profile, as shown in figure 4. Suppose we introduce a uniform distribution of a solute in the flow, shown as the blue rectangle to the left. This ‘plug’ of solute is carried downstream while being sheared out by the flow, distorting the plug as shown. But this shearing of the plug leads to gradients of concentration in the radial direction, and diffusion (indicated by the red arrows) relatively quickly smooths out the radial distribution of the solute over the small radius of the pipe. Thus, we see that, as it moves downstream, the solute spreads out into a broadened, more dilute plug. This plug is much broader than it would be if there were a uniform velocity profile (with no shear): in that case, the initial plug would be carried downstream without distortion and it would broaden only slightly due to diffusion (as shown in figure 4*b*). The effect of the velocity shear can be quantified in terms of an effective lateral diffusion coefficient *D*_eff_. In fact, for the case of pipe Poiseuille flow that we are considering here, there is an analytical solution [44,45] that yields an exact expression for

$$D_{\mathrm{eff}}=D+\frac{a^{2}u_{\max}^{2}}{192D},$$

where *a* is the inner radius of the pipe, *u*_max_ is the maximum flow velocity (at the centre of the pipe) and *D* is the ordinary diffusion coefficient. Taylor dispersion of passive solutes can be included in various flow models by solving the advection–diffusion equation for the computed flow fields.

Something like Taylor dispersion will also occur in the oscillatory flow driven by perivascular pumping. The oscillating shear flow will act along with diffusion to produce enhanced dispersion in the streamwise direction. Asgari *et al.* [22] included this process in their model of perivascular pumping, and they claim that the dispersion is more important than the net flow (which they found to be quite weak). Recently, Sharp *et al.* [46] modelled dispersion in a purely oscillatory shear flow in a PVS. The basic mechanism is illustrated in figure 4*c*. The oscillating shear flow spreads out an initial plug of solute in the axial direction and radial diffusion smooths out the distribution of the solute. However, the advective spreading of the solute in the axial direction occurs only over a finite distance, determined by the frequency and maximum velocity of the shear flow, and any further spreading in the axial direction occurs by diffusion alone. This is in sharp contrast to the case of Poiseuille flow, in which the shear continues to spread out the plug of solute monotonically as it is advected downstream. In the absence of any net flow, the enhanced dispersion due to an oscillatory shear flow will only serve to smooth out the distribution of a solute within the PVS more quickly than if the fluid were at rest, and hence will not serve alone as an effective clearance mechanism.

We should also point out that, in actual PVSs, the solutes of interest (metabolic proteins, for example) will likely not be entering as localized plugs of concentration, as in the illustrations in figure 4, but instead will be entering rather uniformly all along the axial direction, so dispersion resulting from any form of shear flow (steady or oscillatory) will not substantially increase the rate of clearance: the distribution of solute entering the PVS will already be fairly uniform along the axis of the PVS, and diffusion will quickly smooth out the distribution across the small radial distance. The presence of a bulk flow, however, will likely have a significant effect on clearance rate.

For the shearing flow driven by perivascular pumping, there will be both kinds of Taylor dispersion discussed above. The flow consists of a superposition of a purely oscillatory component, leading to limited Taylor dispersion, and a weaker average (bulk) flow in the direction of the wall wave, which at low dynamic Reynolds number will have a velocity profile identical to the steady Poiseuille profile for the particular shape of the PVS annulus. The bulk flow component will provide both Taylor dispersion of the solute and downstream advection, thereby constituting an effective clearance mechanism.

### 6.3. Advection and diffusion in adjacent spaces

It is not clear whether the flow of CSF extends to a flow of ISF throughout the entire brain parenchyma. As mentioned, the continuity equation requires that the net flow of fluid observed in periarterial spaces must continue in some form in other parts of the brain, and there is substantial experimental evidence that it continues as a flow of ISF in parts of the parenchyma [43]. Here, I simply point out that the flow need not reach every part of the parenchyma in order to form an effective clearance mechanism. Advection and diffusion can work together to clear a solute even when they do not occur in the same place.

**Figure 5.**
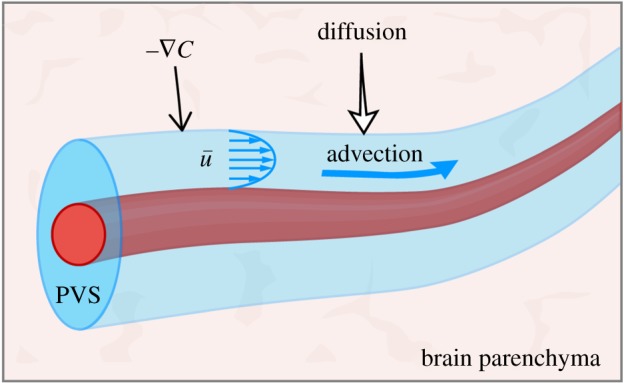
Diffusion into a perivascular flow channel. Advection of the solute downstream by the mean flow of CSF in the PVS maintains the gradient ∇C that drives diffusion of the solute into the PVS. (Online version in colour.)

Consider the configuration shown in figure 5, in which a narrow annular tube with a permeable wall, representing a PVS, passes through a porous space, representing the surrounding brain parenchyma. The PVS is filled with CSF and the spaces in the parenchyma contain ISF. There may be a flow of fluid across the PVS wall through aquaporin-4 channels, but let us ignore that flow in this illustration. First, suppose that the fluids in both spaces are at rest, and that initially there is a uniform concentration *C* of a solute in the parenchyma but no solute in the PVS. If the solute is free to diffuse across the permeable wall of the PVS, with diffusion coefficient *D*, then after a short time of order *τ* = ℓ^2^/*D* (the diffusion time), where ℓ is the width of the PVS, there will be a uniform concentration *C*_1_ of solute throughout the fluid in both the parenchyma and the PVS. If the volume of parenchyma is much greater than the volume inside the PVS, *C*_1_ will be only slightly less than the initial concentration *C*. Moreover, once this new uniform concentration is reached, diffusion will no longer provide any net transport of the solute, and hence the clearance of the solute is not effectively enhanced. Now imagine that the fluid in the PVS is not at rest, but instead is flowing along the PVS at a steady average rate and enters the PVS with zero concentration of the solute. As solute diffuses into the PVS from the surrounding parenchyma, it is swept downstream by the flow; if the flow is fast enough, it effectively keeps the concentration very low at the wall of the tube, maintaining a sharp gradient of *C* at the wall and thus allowing diffusion of solute from the parenchyma to continue. (A recent model of solute transport in the brain interstitium uses this argument to justify applying the boundary condition *C* = 0 at the boundaries of the vasculature [47].) If no new solute is being produced in the parenchyma, then after a sufficient time the solute will be entirely removed from the system. If the solute is being produced in the parenchyma at a steady rate, then a steady-state distribution will be reached and there will be no long-term accumulation. Hence, this process forms an effective clearance system. It may well be that the branching PVS channels threading the brain parenchyma are sufficiently dense such that solutes need only diffuse a short distance to reach a flow channel and be carried away by the flow for disposal. In a sense, this is analogous to the dense arrangement of capillaries that enables sufficiently rapid transport of oxygen to the brain parenchyma, the final step being diffusion over short distances.

## 7. Conclusion

Recent experimental results have disclosed many details of the pulsatile flow of CSF in the PVSs around pial arteries, helping to settle several important, controversial points, and paving the way for improved theoretical modelling of this flow. Previous models of perivascular pumping by arterial pulsations are generally consistent with the experimental results, but there is a need for improved models that account for new findings concerning the size and shape of PVSs, the hydraulic resistance of the PVS and the non-sinusoidal form of the arterial wall wave. There is a need for new experimental techniques that will enable measurements of flow in PVSs around penetrating arteries and other parts of the brain vasculature. Simple hydraulic network models of the entire glymphatic system might be brought into agreement with the experimental flow measurements by incorporating more realistic hydraulic resistances of the PVSs. In this review, I have discussed some basic principles of fluid dynamics and diffusion relevant to flows of CSF, in the hope of convincing a wider readership of the value of fluid-dynamic modelling in understanding the brain’s waste clearance system. An ambitious, long-term goal is to develop a fluid-dynamic model of the entire system with predictive power, to examine the possible effects of abnormalities or interventions. This is a formidable task, and no doubt will take many years, but in the meantime we will learn a lot from simpler models that address specific questions.

## Endnote

1 By contrast, for flows of CSF along the spinal canal the Reynolds number is of the order of 200 and inertial effects cannot be neglected.

## Data accessibility

This article does not contain any additional data.

## Disclaimer

The views and conclusions contained in this review are solely those of the author and should not be interpreted as representing the official policies, either expressed or implied, of the National Institutes of Health, the Army Research Office, or the US Government. The US Government is authorized to reproduce and distribute reprints for Government purposes notwithstanding any copyright notation herein.
